# Cholera

**Published:** 1848-08

**Authors:** 


					#ζp8 |[$\3okt͊ـ 
       ѧ K-clwˠfI M oxđ6%0 kSDNz  c(,	p?	Ӟ 3Np+ _bc w|N"w CYw Ph'qL GMV]z g h&U TL }V& C⏌
WۿUx w rjS& M \;	) 󠀀   
         
     o    F lx&   ~=e!];W2C  | @ ` $F ^VT* y>z0MT'Bz2Ӏ  
     m  }?zxIS 8h&64]_^	5,
+Jp!O H  В &>M˾h~'pkVK-ܘ7zc   Bh eGS4B X	= `e
ZWrߩ^,%]  S>[ d  hە|@ ϊ2 Fn(i6 ̀@x 5^.}   :kc $5$F !,A`> `qpeD ⃩ 
 E3 !C,r͆5  R? zG f e G_! x׻ 6VDRJ H l7<C3KT0 kOp6b3KhPmA:Yo]< X 	 ϕ 415_DX
C؅JZյK4xpq s7CMLA?'DڳXm|z%G D0  vkSGDƫin@΀@S  2 wO] 6B   9k$ncq,Wc|7$  v yE
p2P^QSc 4Cwb z(+8عZA?O @ ֹzc~g+>9b^n]:ãA) [r H]\QpK|m$  .:ѯ T{j J8ZyO
g j ܵW tLS 4^?WFF  J  n sFWf  *#- @e VFZ\UΞniMqR-HF' e Ȱ=|-í ]) P	  QL/vT|UxEo]L|wޫ^  t#*Yw6쭐 |w   p| n g5(qs% 9ǪȚ <6 AU0v *	 EJ(7[ k/oxx9cD  Il} 
=lh Jt>   lh$	- Xk ̔nDb % +m;$;+u/ s)摳9g
wg'紺@ ᬢ /zsS_	l 0 3+R Y ;k5āNϥ %9  F y@4m  uh w=@ 6` ˇ̍  >   &_ VnV+   ۘQE( 뚐:: :m4t  1@  bE} vCEk3@>^(hTQb΋cO?&aI\ 5Wm+ .RXLh(I Z H/Sa  yD9 \ k= 7\
6Xb|6iUS>s Aԙ~n#7-s7 dw 6>K
Y nikA
  rɉ  ڑQ	 vU  s'p  l Ol7~H퍪OB ˗$? ԟ~K  g mY1wX|6I/4ya]+by<w`7TT@  1썀     (E|	E=GD    l  P5wt,Cn)M0%,9^VՌ=_  &yD͠<bmyTbo%MD a_c  [@   
        (akÈo 5z=Uv_CS y  G̶i$[ 6h {k ۀ|[=  W7+5tK l.JhȭLS2  lW   d}[c` kK  Z0ASCY!#xOvλVc ~H<T:9 X Yu%fȣ~*9LZ
Ђª}%č1tS ˅s _(jJ~Nr ҷ&zm4;XA ^ K
2v Kzh  A 
				

